# A novel sialylation pathway mediated by extracellular vesicles in aggressive prostate cancer

**DOI:** 10.1371/journal.pone.0329014

**Published:** 2025-09-12

**Authors:** Camila A. Bach, Md Niamat Hossain, Ishan J. Chaudhari, Cecilia E. Verrillo, Nicole M. Naranjo, Isabella Amoroso, Anna Testa, Samuel Sey, William K. Kelly, Susan L. Bellis, Aurelio Lorico, Ada G. Blidner, Gabriel A. Rabinovich, Lucia R. Languino

**Affiliations:** 1 Prostate Cancer Discovery and Development Program, Thomas Jefferson University, Philadelphia, Pennsylvania, United States of America; 2 Department of Pharmacology, Physiology, and Cancer Biology, Thomas Jefferson University, Philadelphia, Pennsylvania, United States of America; 3 Laboratorio de Glicomedicina, Instituto de Biología y Medicina Experimental, Consejo Nacional de Investigaciones Científicas y Técnicas, Buenos Aires, Argentina; 4 Facultad de Ciencias Exactas y Naturales, Universidad de Buenos Aires, Ciudad de Buenos Aires, Argentina; 5 Department of Medical Oncology, Thomas Jefferson University, Philadelphia, Pennsylvania, United States of America; 6 Department of Cell, Developmental and Integrative Biology, University of Alabama, Birmingham, Alabama, United States of America; 7 Department of Basic Sciences, College of Medicine, Touro University, Henderson, Nevada, United States of America; 8 CaixaReseach Institute, Barcelona, Spain; Universita degli Studi Gabriele d'Annunzio Chieti e Pescara, Italy

## Abstract

Altered cell surface glycosylation is a hallmark of cancer; among aberrant glycan structures, hypersialylated proteins contribute to disease progression. The enzyme ST6 β-galactoside α2,6-sialyltransferase 1 (ST6GAL1) mediates α2,6-linked sialylation of N-glycosylated proteins and is upregulated in many cancers, including prostate cancer (PrCa). We propose that ST6GAL1 may be released by cancer cells in small extracellular vesicles (sEVs) in the PrCa tumor microenvironment to potentially modulate cell surface sialylation in recipient cells. We isolated sEVs from PrCa cells by density gradient separation and characterized them by nanoparticle tracking analysis using ZetaView and immunoblotting analysis. We identified ST6GAL1 in both its membrane-bound and soluble forms, both active, in circulating sEVs from healthy donors and patients with PrCa. ST6GAL1 is also expressed in human PrCa cells (PC3, DU145, and C4-2B), and in murine cells (TRAMP-C2 and RM1) at different levels, which correlate with aggressive cell phenotypes. In addition to classic sEV markers, such as CD9, TSG101 and Syntenin, sEVs isolated from PrCa cell lines express PDL1, an immune checkpoint ligand. The soluble ST6GAL1 form is present in the sEVs released from DU145 and PC3 cells and can be transferred via sEVs to recipient PrCa cells. This transfer is prevented by expression of Nogo-66 receptor homolog 2 (NgR2) and β3 integrin, which are elevated in the aggressive neuroendocrine phenotype of the disease. The soluble form is absent in the sEVs released from the bone metastatic line C4-2B, which only contains the membrane-bound form. Our results suggest that ST6GAL1 in sEVs derived from PrCa cells may potentially play a role in promoting bone metastasis by facilitating the formation of the pre-metastatic niche.

## Introduction

Hypersialylation, defined as elevated levels of glycans containing sialic acid, is a common feature of cancer cells, driving disease progression and facilitating immune evasion, therefore contributing to establishing an immunosuppressive tumor microenvironment (TME) [1]. Genetic and enzymatic desialylation using neuraminidases in tumor cells delays tumor growth in murine models and induces the repolarization of tumor-associated macrophages, enhancing antitumor immunity and immune checkpoint inhibitors’ efficacy [1].

Sialyltransferases are key enzymes in the biosynthetic pathway of glycans containing sialic acid [2]. This subset of glycosyltransferases catalyzes the transfer of sialic acid from cytidine monophosphate (CMP) to the terminal ends of carbohydrate chains attached to proteins or lipids. The most well-described sialyltransferase in humans is ST6 β-galactoside α2,6-sialyltransferase 1 (ST6GAL1), a transmembrane protein that mediates the α2,6-sialylation of N-glycosylated proteins [3]. ST6GAL1 expression is low in normal tissue but increases in many cancer cell types [3,4].

Studies performed in animal models suggest that ST6GAL1 promotes cell migration and invasion, and *in vitro* studies indicate that this may be due to its role in mediating the sialylation of the β1 integrins [4,5]. Bresalier et al. [6] observed that metastatic murine cell lines exhibit higher sialylation levels than their less aggressive parental lines, and treatment with neuraminidase drastically reduces the number of liver metastases in these metastatic lines. Similarly, metastatic clones isolated from human tumor cell lines show increased ST6GAL1 expression compared to the parental population, and ST6GAL1 knockdown in the metastatic clones impairs primary tumor growth and metastasis [7]. Changes in α2,6 sialylation are also evident within immune and endothelial compartments in settings of immunosuppression and aberrant angiogenesis [8,9]. Additionally, ST6GAL1 regulates the ability to evade cell death and has been established as a key negative regulator of apoptosis, which depends on a family of proteins called Galectins [5]. For example, Galectin-3, a member of this family, binds directly to β1 integrins and induces apoptosis, but only when the β1 integrin subunit lacks α2,6-sialylation [10].

In prostate cancer (PrCa), ST6GAL1 levels are elevated in patient plasma [11], and ST6GAL1 expression positively correlates with Gleason score, seminal vesicle involvement, and poor survival [12]. Both *in vitro* and *in vivo* studies have demonstrated that ST6GAL1 promotes tumor growth and invasion. In aggressive PrCa cell lines, DU145 and PC3, silencing the ST6GAL1 gene reduces cell proliferation, migration, and invasion, leading to decreased PI3K/Akt signaling pathway activity [12]. Additionally, Hodgson et al. [13] reported that ST6GAL1 is particularly elevated in PrCa that has metastasized to bone compared to lymph nodes or the primary tumor.

In addition to its membrane-bound form, ST6GAL1 can also be found in a soluble form [14,15]. This variant arises from proteolytic cleavage, which removes the transmembrane and cytosolic domains of ST6GAL1, leaving the luminal domain containing the catalytic site intact. β-secretase 1 (BACE1) is the primary enzyme responsible for this cleavage [16]. The soluble form of ST6GAL1, with a molecular weight of 35–45 kDa, can be secreted into the extracellular space, thus promoting the remodeling of glycans. This “extracellular” sialylation has been more extensively studied in the context of the hematopoietic system [17]. ST6GAL1 has been found in rodent serum, mainly released by the liver during acute-phase reactions [18,19], and it has been described that serum glycosyltransferases can participate in extracellular glycosylation reactions [20]. On the other hand, although CMP-sialic acid is not typically found outside the cells, activated platelets release CMP-sialic acid into the circulation, allowing ST6GAL1 to sialylate proteins in serum and on the cell surfaces [21]. In addition to liver tissue, platelets appear to be a relevant source of ST6GAL1 [21,22].

Manhardt et al. [22] observed that ST6GAL1-deficient cells isolated from the bone marrow of chimeric mice acquired a positive phenotype for *Sambucus Nigra* agglutinin (SNA), a lectin that binds specifically to α2,6 sialic acid, when transferred into competent mice. Similar studies, including the administration of recombinant soluble ST6GAL1, have reported extracellular sialylation mediated by ST6GAL1 and its physiological relevance in monocyte/macrophage differentiation [23]. Additionally, B cells have been shown to release active ST6GAL1, which modifies hematopoietic progenitor cell surface glycosylation, therefore suppressing granulopoiesis [24], a previously established effect of extracellular ST6GAL1 [25]. However, these studies do not address the nature of the extracellularly sialylated molecules.

A recent study suggests that ST6GAL1 secreted by breast cancer cells in soluble form or as a membrane-bound form within extracellular vesicles (EVs) could complement its intrinsic activity in tumor cells, remodeling surface glycans and promoting tumorigenesis and invasiveness [15]. The membrane-bound form of ST6GAL1 detected in small EVs (sEVs) could explain previous observations of this full-length 50 kDa ST6GAL1 form detected in the media of lymphoblastic cell lines [24]. Moreover, BACE1-cleaved and membrane-bound forms of functional ST6GAL1 are carried in sEVs and particles, such as exosomes and exomeres, derived from colorectal cancer cell lines [26]. This functional ST6GAL1 can be transferred to recipient cells, resulting in the hypersialylation of cell surface proteins such as the β1 integrins [26]. The release in EVs represents an additional potential source of soluble glycosyltransferases in the extracellular space.

Here, we analyze the expression of ST6GAL1 in its soluble and membrane-bound form in sEVs released by PrCa cells, and its transfer by sEVs, which has a potential influence on tropism and metastatic progression.

## Materials and methods

### Human plasma

Plasma samples from eight patients with PrCa (Table 1) and three healthy male donors were obtained at Thomas Jefferson University to isolate sEVs. The study was conducted in compliance with the Declaration of Helsinki and approved by the Institutional Review Board (IRB) of Thomas Jefferson University (Protocol 19D.011). Written informed consent was obtained from all subjects involved in the study. The specimens were de-identified and discarded following the IRB of Thomas Jefferson University guidelines. Following are the dates for each individual patient’s blood collection: Sample ID # A (6/25/2024); B (10/3/2024); C (12/16/2024); D (12/17/2024); E (12/17/2024); F (6/6/2024); G (8/16/2024); H (8/27/2024).

**Table 1:** Pathological characteristics, Gleason grades and protein (including sEV marker) expression in sEVs from patients with PrCa. Prep# 1 is shown in the top panels of Fig 2A; Prep# 2 is shown in the bottom panels of Fig 2A.

Prep#	ID #	PSA	Pathological Stage	Grade Group	Gleason Score	Metastasis	CD9	NgR2 and β3	Syntenin
**1**	A	42.6	T3bN1	5	4 + 5 = 9	Yes	+	+	+
	B	4.2	T3bN1M0	5	4 + 5 = 9	Yes			
	C	3.1	T3bN0	3	4 + 3 = 7 and 3 + 3 = 6, (more than one tumor focus)	No			
	D	4.6	T3aN0	2	3 + 4 = 7	No			
	E	4.9	T2N0M0	2	3 + 4 = 7	No			
**2**	F	2.9	T2N0	2	3 + 4 = 7	No	+	+	+
	G	4.5	T2N0	2	3 + 4 = 7	No			
	H	7.2	T2N0M0	2	3 + 4 = 7	No			

### Cell culture

Human prostate adenocarcinoma cell lines PC3 (CRL-1435, RRID:CVCL_B0E3), DU145 (HTB-81, RRID:CVCL_0105), and C4-2B (CRL-3315, RRID:CVCL_4784) were obtained from ATCC and cultured as previously described [27]. Additionally, murine prostate adenocarcinoma cell lines TRAMP-C2 (ATCC, CRL-2731, RRID:CVCL_3615) and RM1 (provided by Dr.T.Thompson, Baylor College of Medicine, Houston, Texas, USA, RRID: CVCL_B459), and the mouse fibroblast cell line NIH3T3 (ATCC, CRL-1658, RRID: CVCL_0594) were used. All murine lines were cultured in DMEM medium (Corning, 10–013-CV) supplemented with 10% fetal bovine serum (FBS) (R&D Systems, S11550) and 1% Penicillin/Streptomycin (Gibco, 15140–122). The medium for TRAMP-C2 cells was also supplemented with 0.005 mg/mL of human insulin (Sigma-Aldrich, I9278) and 10 nM dehydroisoandrosterone. Cells were seeded in sterile 150 mm culture plates (Fisher Scientific, FB012925) and maintained in a humidified incubator at 37°C and 5% CO_2_. Subculturing was performed with 0.05% trypsin 0.53 mM EDTA (Corning, 25–052-CI) at 37°C for 2–5 minutes.

DU145 cells were transfected with a vector carrying *RTN4RL2* cDNA, encoding for Nogo-66 receptor homolog 2 (NgR2) or an empty vector (Origene, SC310413 and PS100001, respectively), named as NgR2 transfectants or Mock DU145 cells, as previously described [28].

### Isolation of EVs by differential ultracentrifugation

The EVs derived from PrCa cells (PC3, DU145, and C4-2B) and human plasma of patients with PrCa and healthy donors were isolated as previously described [29,30]. The pellets from ultracentrifugation containing the EVs were resuspended in PBS. This process was repeated three times. The EV preparations were pooled and centrifuged at 100,000 x *g* for 70 minutes at 4°C. The resulting pellets were resuspended in 60–150 µL of PBS for sEV isolation.

### sEV isolation by iodixanol density gradient separation

sEV isolation by iodixanol density gradient separation (IDG) was performed as previously described [30,31].

### Immunoblotting

A robust cell lysis was ensured for the immunoblotting (IB) detection of ST6GAL1. Cells were kept on ice for 10 minutes with lysis buffer (10 mM Tris-HCl, pH 7.4, 150 mM NaCl, 1 mM EDTA, 0.1% SDS, 1% Triton X-100 and 1% sodium deoxycholate, supplemented with the protease inhibitors calpain, aprotinin, leupeptin, pepstatin, sodium orthovanadate and sodium fluoride) before being scraped from the plate. The sample was passed through a 1 mL insulin syringe to break up the cell fragments further while carefully avoiding bubbles and then kept on ice for 15 minutes with vortexing every 5 minutes. The samples were centrifuged at 13,200 rpm (16,363 x *g*) at 4°C for 30 minutes. For sEV samples, the lysis buffer was added during the sample preparation before loading onto the gel. Protein quantification was performed using the DC™ Protein Assay kit (Bio-Rad, 5000113, 5000114, and 5000155) following the manufacturer’s instructions.

All samples were prepared with 4X reducing Laemmli buffer (0.25 M Tris-Cl, pH 6.8, 8% SDS w/v, 0.4% bromophenol blue w/v, 40% glycerol v/v, 6% β-mercaptoethanol). Between 60–85 µg of total cell lysate (TCL) and 10–17 µg of sEV protein were loaded and separated onto a 10% SDS-PAGE. The gel was run overnight at 9–13 mA. Transfer was performed for 6 hours at 38 mA in a cold room at 4°C, using PVDF membranes (0.45 µm pore size, Millipore, IPFL00010) that were pre-activated in methanol for 1–2 minutes.

The membranes were incubated with blocking buffer (5% non-fat dry milk in Tris-buffered saline (TBS) with 0.15% Tween 20 (TBS-T)) overnight at 4°C. Afterward, three 10-minute washes with TBS-T were performed. For ST6GAL1 detection, the membranes were then incubated for 48 hours at 4°C with 1 µg/mL of primary antibody against ST6GAL1 (R&D Systems, AF5924) in blocking buffer. Then, the membranes were washed three times in TBS-T for 10 minutes and incubated with anti-goat IgG-HRP secondary antibody (R&D Systems, HAF109) at a 1:2000 dilution in 5% milk in TBS-T for 1 hour at room temperature. Three 10-minute washes with TBS-T were performed. Finally, the membranes were developed using WesternBright™ ECL or Sirius HRP substrate kits (Advansta Inc., K-12045-D50 or K-12043-D10, respectively).

Other primary antibodies were incubated overnight. Rabbit monoclonal antibodies to: β-Actin (Cell Signaling, 4970, RRID:AB_2223172) 1:1000, Tumor susceptibility gene 101 (TSG101) (Abcam, ab125011, RRID:AB_10974262) 1:1000, β3 integrin (Cell Signaling, 13166S, RRID:AB_2798136) 1:1000, Syntenin (Abcam, ab133267, RRID:AB_11160262) 1:1000, Programmed cell death ligand 1 (PDL1) (Cell Signaling, 13684, RRID: AB_2687655) 1:1000 and CD41 (Abcam, ab134131, RRID:AB_2732852) 1:1000 were used. Rabbit polyclonal antibodies to: Calnexin (Cell Signaling, 2433, RRID:AB_2243887) 1:1000 and total focal adhesion kinase (tFAK) (Santa Cruz, sc-558, RRID:AB_2300502) 1:250 were used. Mouse monoclonal antibodies to: CD9 (Santa Cruz, sc-13118, RRID:AB_627213) 1:200 and CD81 (Abcam, ab23505, RRID:AB_447487) 1:1000 were used.

### ZetaView nanoparticle tracking analysis

The ZetaView QUATT equipment (Particle Metrix, RRID:SCR_016647) was used to measure the size distribution and concentration of sEVs. Samples were diluted in double distilled water, and 1.5 mL of the dilution was used for the analysis. The ideal concentration was determined by pre-testing serial dilutions from a 1:1000 dilution of the sample to ensure the number of particles per frame was between 140–200 and the measured concentration was in the order of 10⁷ particles/mL. For each measurement, 11 positions were scanned, and 60 frames per position were captured (video settings: high) with a 488 nm laser and the following settings: focus: autofocus, scatter filter wavelength, camera sensitivity at 80, shutter at 100, minimum brightness at 20 and cell temperature at 23°C.

### ST6GAL1 transfer via sEVs to PrCa cells

TRAMP-C2 and DU145 cells were used as recipients and seeded at concentrations of 2 × 10⁵ and 3 × 10⁵ cells/well, respectively, in 6-well plates and incubated (37°C, 5% CO₂) in DMEM with FBS and supplemented as indicated above for 24 hours and then washed with PBS. TRAMP-C2 cells were incubated with 5 µg/mL of sEVs derived from PC3 cells, while DU145 cells were incubated with 10 µg/mL of sEVs derived from NgR2 transfectant [32] or Mock DU145 cells resuspended in a medium without FBS. PBS was used as a negative control. After 24 hours of incubation, the cells were washed three times with PBS, then harvested by scraping and analyzed by IB.

## Results

### ST6GAL1 membrane-bound form in sEVs from patients with PrCa associates with an invasive phenotype

**Fig 1:**
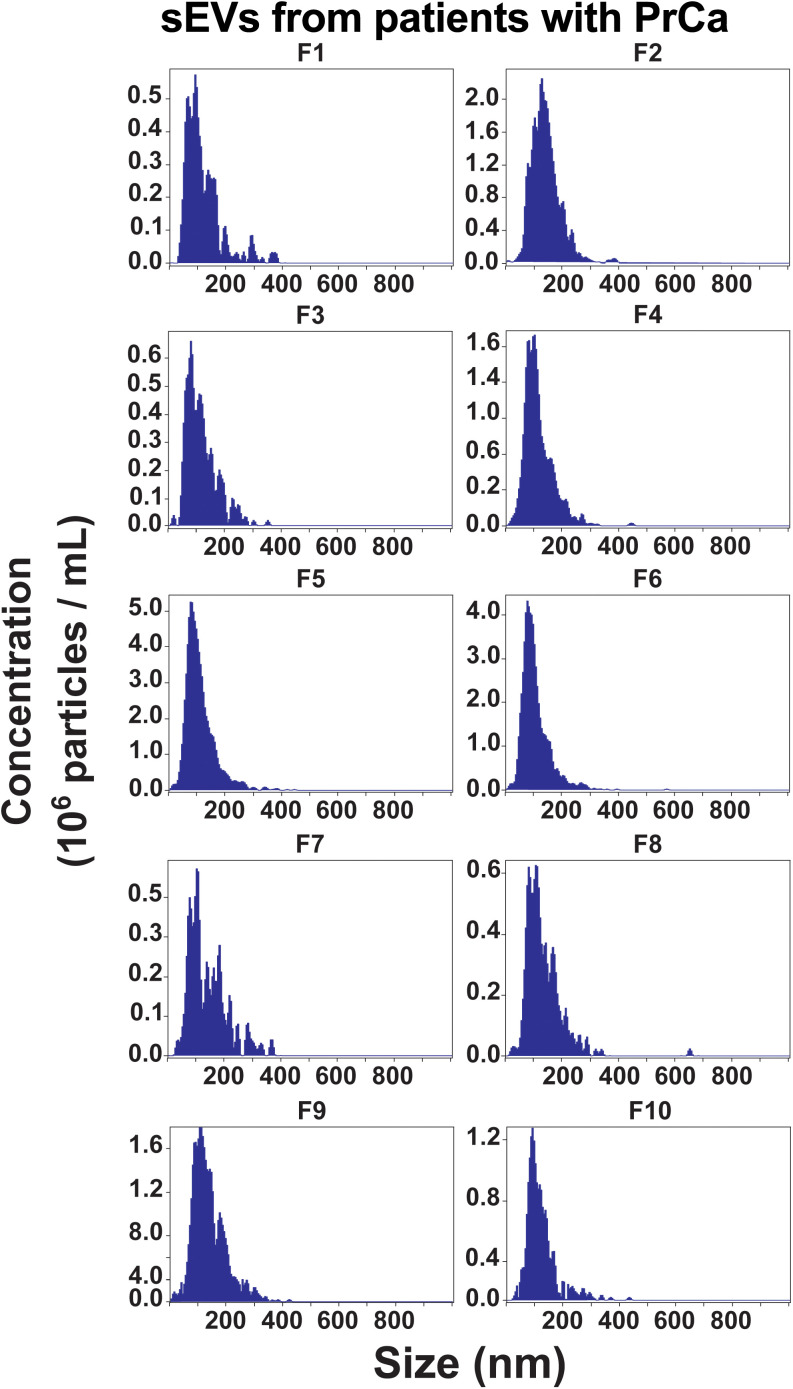
Size distribution analysis of small extracellular vesicles (sEVs) from patients with invasive prostate cancer (PrCa) by ZetaView. Fractions 1-10 were analyzed using ZetaView after iodixanol density gradient (IDG) separation.

To assess the distribution of circulating ST6GAL1 forms and their association with tumoral progression, we isolated sEVs from the plasma of patients with PrCa by IDG separation. This study included two groups of patients with PrCa, and the characteristics of their pathology are detailed in Table 1. The first group (Prep# 1) exhibits an invasive phenotype of the disease consisting of five cases with Gleason scores 7 and 9 and pathological stages T3bN1, T3bN1M0, T3bN0, T3aN0 and T2N0M0, who presented seminal vesicle invasion and/or regional lymph node spread. The second group (Prep# 2) consists of a noninvasive homogenous group including three patients with Gleason score 7 and stage T2N0 and T2N0M0, where the tumor was confined to the prostate without regional or distant metastases. Size distribution analysis of circulating sEVs from patients with invasive PrCa, Prep# 1, using ZetaView shows that the size of sEVs falls below 200 nm (Fig 1) like all the sEVs analyzed in this study.

**Fig 2:**
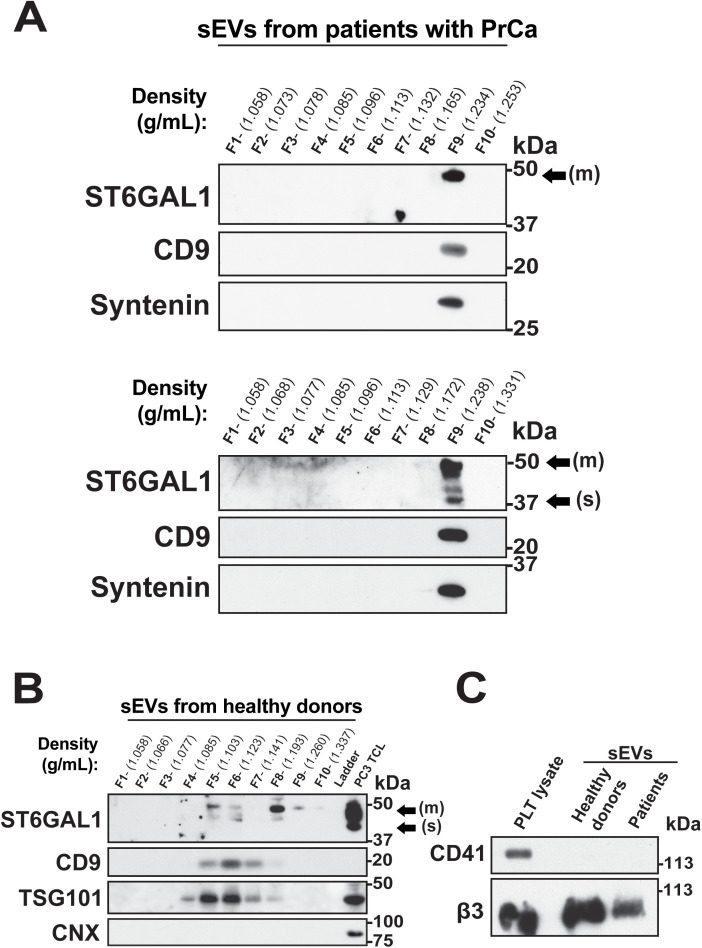
ST6GAL1 expression in sEVs from plasma of patients with PrCa. **(A)** Immunoblotting (IB) analysis of ST6GAL1, CD9 and Syntenin in lysates of sEVs isolated by IDG separation from plasma of patients with invasive (top, Prep# 1) or noninvasive (bottom, Prep# 2) PrCa; the total volume (30 μL) of each fraction was used. **(B)** IB analysis of ST6GAL1, CD9, TSG101 and Calnexin (CNX) in sEVs from plasma of healthy donors isolated by IDG and in PC3 total cell lysate (TCL); the total volume of each fraction and 40 µg of PC3 TCL were used. **(C)** IB analysis of CD41 and the αVβ3 integrin (β3) in platelet (PLT) lysate and sEVs from the plasma of healthy donors and patients with PrCa; 10 µg of TCL and 20 µg of sEV lysates were used. (m) indicates the membrane-bound ST6GAL1 form and (s) the soluble form.

We assessed the expression of classical sEV markers CD9 and Syntenin in sEVs derived from the plasma of patients with PrCa via IB analysis and found that both markers are expressed in fraction 9 of the gradients (Fig 2A). Fraction 9 in both groups exhibits a density of 1.23-1.24 g/mL, which is higher than the typically reported density for sEVs [33], but consistent with prior observations for sEVs derived from human plasma and saliva [34,35]. IB analysis shows that sEVs from both groups of patients contain the αVβ3 integrin (β3) and its downstream effector NgR2, a marker of neuroendocrine PrCa (Table 1). We show that ST6GAL1 exclusively co-fractionates with the sEV markers in fraction 9 in both groups and is not found in fractions where classical sEV markers are not detected (Fig 2A). While sEVs derived from both groups of patients carry the membrane-bound form of ST6GAL1, only the sEVs from the noninvasive group carry the soluble form. Hence, although all analyzed proteins are consistently expressed in sEVs from both the invasive and noninvasive groups of patients, we observe differential ST6GAL1 isoform expression.

To determine differences with healthy individuals, we analyzed sEVs derived from the plasma of male donors without known prostate pathology. The characteristic markers of sEVs, either CD9 or TSG101, were detected in healthy controls in fractions with a density range (∼1.08–1.19 g/mL) lower than in fractions from patients with PrCa (∼1.23-1.24 g/mL) (Fig 2B). In contrast to the focal distribution observed in patients, sEV markers in plasma from healthy donors appear widely spread in fractions 4–8 of the gradient. Both forms of ST6GAL1 are identified in healthy donors, and the membrane-bound form shows a dual localization pattern in the sEV fractions 5/6, and in fraction 8 as well as in fractions 9 and 10, where classical EV markers are not detected. As Kowal et al. [36] reported for sEV markers, we observed a bimodal distribution of ST6GAL1. The absence of Calnexin (CNX) in the sEV lysates indicates that the samples are free from contamination by components of the endoplasmic reticulum compartment.

Since ST6GAL1 and its substrate CMP-sialic acid can be released from platelets [21,22], we confirmed that our sEV isolation protocol from human plasma is platelet-free by assessing the expression of CD41, a marker of platelets and their precursors, by IB (Fig 2C). Both preparations from healthy donors and patients with PrCa are negative for CD41, whereas the platelet lysate is positive. Thus, the differential distribution of ST6GAL1 membrane-bound form in sEVs according to tumor aggressiveness suggests its potential as a biomarker of progression in PrCa.

### ST6GAL1 form-specific distribution in sEVs released from human PrCa cells with different metastatic potential

**Fig 3:**
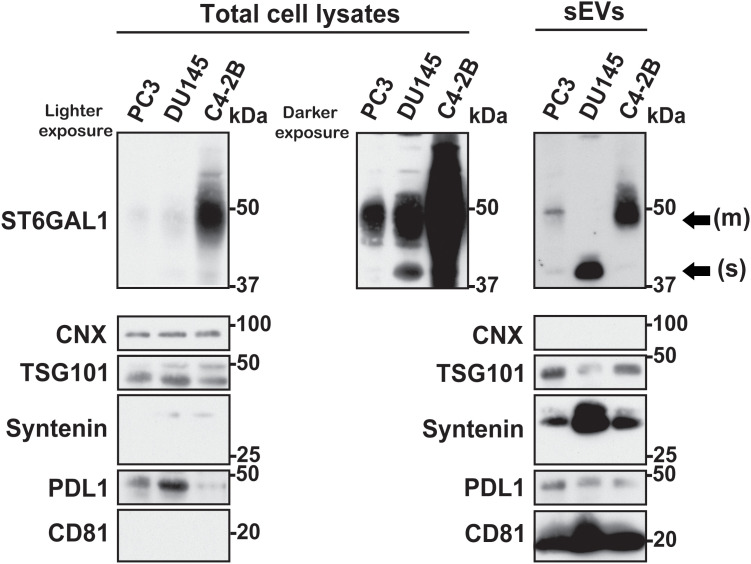
ST6GAL1 expression in human PrCa cell lines and their derived sEVs. IB analysis of ST6GAL1 at lighter (left panel) and darker exposures (middle panel), CNX, TSG101, Syntenin, PDL1 and CD81 in PC3, DU145 and C4-2B TCL and sEVs isolated by IDG separation (fractions 1-5 pooled; right panel); 85 µg of TCLs and 17 µg of sEV lysates were used.

Given the suggested role of ST6GAL1 in cancer progression, we assessed its expression in the human PrCa cell lines PC3, DU145 and C4-2B, which portray a range of disease progression and their derived sEVs (Fig 3). With even loading evidenced by CNX and TSG101, IB analysis reveals ST6GAL1 is increased in C4-2B cells [37]. The sEVs express the classical markers TSG101, CD81, and Syntenin. Additionally, the absence of CNX in the lysates from sEVs indicates that the samples analyzed are free from contamination by components of the endoplasmic reticulum. IB analysis demonstrates that sEVs derived from PC3 cells retain low levels of both the membrane-bound and soluble forms of ST6GAL1. sEVs derived from DU145 cells, which were first isolated from brain metastasis and do not metastasize to bone, only contain the soluble form of ST6GAL1. In contrast, sEVs derived from C4-2B, a cell line which spreads to the bone *in vivo* [37], exclusively retain the membrane-bound form of ST6GAL1. Moreover, the expression of the immune checkpoint ligand PDL1 is lower in C4-2B cells than in PC3 and DU145 cells but is comparable between them on their sEVs, indicating a selective packaging of the cargoes of sEVs. These results show that ST6GAL1 forms are selectively packaged in a cell-type-specific manner, thus suggesting that metastatic tropism may be linked to distinct sialylation delivered via sEVs.

### Differential ST6GAL1 expression patterns in murine PrCa models

**Fig 4:**
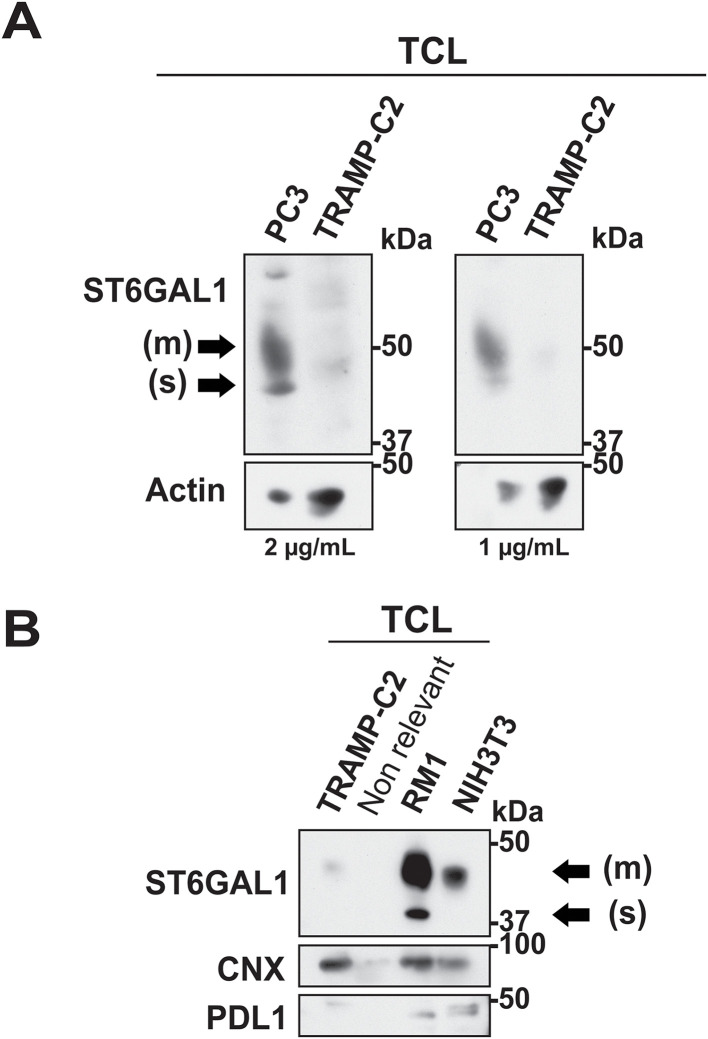
ST6GAL1 expression in murine PrCa cell lines. **(A)** IB analysis of ST6GAL1 and actin in PC3 and TRAMP-C2 TCL using 1 μg/mL (right panel) or 2 μg/mL (left panel) of ST6GAL1 antibody; 40 µg of TCLs were used. **(B)** IB analysis of ST6GAL1, CNX and PDL1 in TRAMP-C2, RM1 and NIH3T3 TCLs; 85 µg of TCLs were used. A lane loaded with non relevant sample is included (Non relevant).

To validate the cross-reactivity of the ST6GAL1 antibody previously described [38], we analyzed ST6GAL1 expression in human (PC3) and murine (TRAMP-C2) PrCa cell lines. Despite higher loading in TRAMP-C2 lysates, evidenced by actin expression, the ST6GAL1 signal is weaker in TRAMP-C2 lysates compared to PC3 lysates (Fig 4A). All the following experiments were conducted employing 1 μg/mL of antibody.

Next, we compared ST6GAL1 expression in the murine PrCa cell lines TRAMP-C2 and RM1 and the murine fibroblast cell line NIH3T3 by IB (Fig 4B). We characterized TRAMP-C2, which grows slower than RM1 cells and requires dihydrotestosterone for *in vitro* growth [39], as a low-level ST6GAL1 expression cell line. Moreover, the fibroblast cell line NIH3T3 predominantly exhibits the membrane-bound form. In contrast, we characterized RM1 cells, an androgen-independent and highly aggressive cell line [39], as a high-level ST6GAL1 expression cell line. Finally, RM1 cells express both forms of the ST6GAL1 enzyme.

### ST6GAL1 transfer via sEVs to PrCa cells

**Fig 5:**
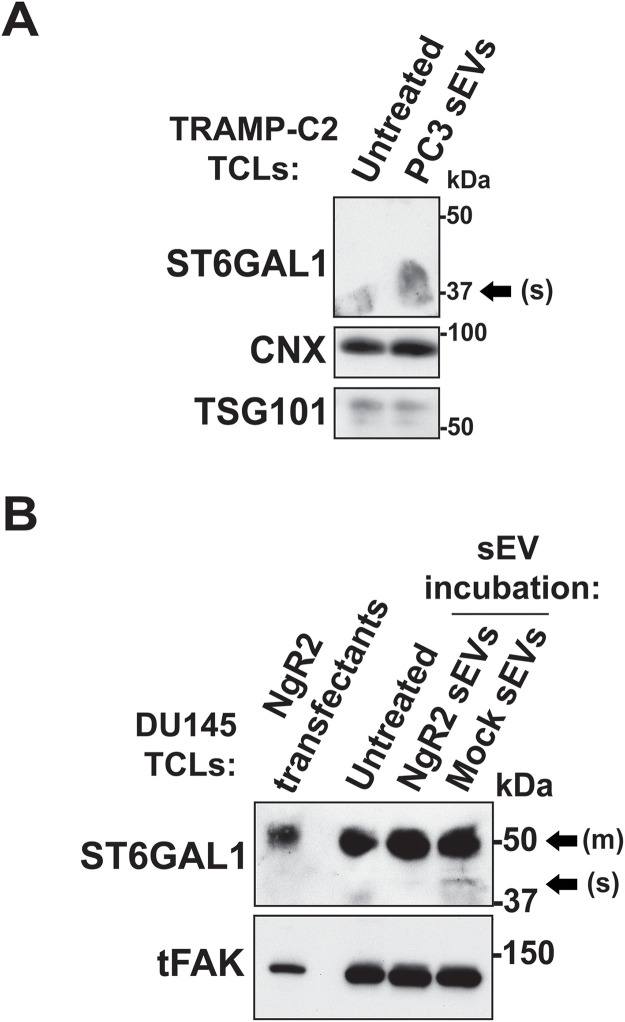
Transfer of ST6GAL1 carrying sEVs to recipient PrCa cells. **(A)** IB analysis of ST6GAL1, CNX and TSG101 in TRAMP-C2 TCL collected 24 hours after incubation with sEVs derived from PC3 cells isolated via IDG or PBS (untreated); 60 µg of TCLs were used. **(B)** IB analysis of ST6GAL1 and total focal adhesion kinase (tFAK) as loading control in DU145 exogenously expressing NgR2 (NgR2 transfectants) TCL, DU145 TCL collected 24 hours after incubation with sEVs from DU145 cells exogenously expressing NgR2 (NgR2 sEVs) or control Mock-DU145 sEVs (Mock sEVs) isolated via IDG or PBS (untreated); 40 µg of TCLs were used.

We investigated whether ST6GAL1 in sEVs from PrCa cells can be transferred to recipient cells. For this purpose, we isolated sEVs via IDG from PC3 cells, which carry both forms of ST6GAL1, and incubated them with TRAMP-C2 cells, selected for their low levels of ST6GAL1 expression. After 24 hours, the TRAMP-C2 cells were lysed and analyzed by IB for ST6GAL1, CNX and TSG101 (Fig 5A). The incubation with sEVs from PC3 cells increases the soluble form of ST6GAL1, whereas the membrane-bound form remains undetectable, confirming the cross-species transfer of ST6GAL1 to recipient cells.

Then, DU145 cells were incubated for 24 hours with sEVs isolated via IDG from DU145 cells exogenously expressing NgR2 (NgR2 sEVs), which promotes their protumorigenic activity [32]. Control sEVs were obtained from mock-transfected DU145 cells (Mock sEVs). TCLs were analyzed by IB for ST6GAL1 and tFAK, used as loading control (Fig 5B). The expression of the soluble form of ST6GAL1 on the recipient cells increases upon incubation with Mock sEVs but not with NgR2 sEVs. This result indicates that NgR2 in sEVs may prevent the cleavage, loading, or subsequent transfer of the soluble form of ST6GAL1 to recipient cells.

## Discussion

The results reported in this study show that ST6GAL1 is expressed in human and mouse PrCa cells and their derived sEVs as well as in circulating sEVs from healthy donors and patients with PrCa. Our data also show that the ST6GAL1 soluble form can be taken up by recipient cells when incubated with sEVs carrying ST6GAL1 and may suggest that this uptake is impaired by NgR2 in the cargo of the sEVs.

This study could bring light into the widely described tumorigenic role of ST6GAL1 in many cancer cell types [2,4–6], including PrCa [11,12], as it contributes to tumor growth and invasion [7] while it is expressed at low levels in the epithelium of normal tissues [4]. ST6GAL1 is a transmembrane protein [3] but can also be detected extracellularly as soluble form; however, the study of the ability of ST6GAL1 soluble form to mediate intracellular signaling, extracellular signaling, tumor growth or invasion remains incomplete. ST6GAL1 potential targets are proteins that are upregulated in metastatic cancer. Among others [40–42], ST6GAL1 sialylates the β1 integrins (as previously described by [10]) and may sialylate the αVβ3 integrin/NgR2 complex, which contributes to differentiation toward an aggressive metastatic neuroendocrine PrCa phenotype [28,32]. Illustrating the relevance of sialylation in this complex, is the evidence that the αVβ3 integrin is sialylated in the α2,6 position [43,44] and NgR2 binds myelin-associated glycoprotein in a sialic acid-dependent manner [45]. Our study shows that the ST6GAL1 soluble form is transferred from human donor cancer cells to (human or murine) recipient cancer cells via sEVs. We also show that the αVβ3 integrin/NgR2 complex in cells prevents sEV transfer of the ST6GAL1 soluble form, indicating that ST6GAL1 soluble form may remain extracellular. While DU145 shows both forms of the enzyme, when they express NgR2 and become more aggressive, they do not exhibit the soluble form. Hence, their sEVs may not carry the soluble form, which cannot be transferred. This can be due to a NgR2 effect or a consequence of a more aggressive phenotype.

Regarding ST6GAL1 role in organ-site specific metastatic behavior, a recent study demonstrates that sEVs derived from breast cancer cells show increased α2,6 sialylation compared to their parental cells, and this increase is higher in the variant that metastasizes to the bone than in the one that metastasizes to the lung [46]. Moreover, another recent report shows that sialic acid blockade inhibits the metastatic spread of PrCa to bone [13]. In line with these findings, we show that ST6GAL1 is enriched in bone metastatic C4-2B cells and sEVs; we therefore suggest that vesicular ST6GAL1 may have a role in promoting bone metastatic behavior in PrCa as it may promote pre-metastatic niches as suggested by Hait et al. [15]. The ST6GAL1 membrane-bound form is packed in sEVs from bone metastatic C4-2B cells but not from DU145 cells, which do not metastasize to bone; we thus propose that the ST6GAL1 membrane-bound form in sEVs may help the donor cancer cells to promote bone metastasis by targeting local cells, as osteoblasts or osteoclasts [47]. While our study demonstrates a variation in ST6GAL1 expression among human and mouse PrCa cell lines, potentially correlating with their reported aggressive phenotypes, these cell lines have been maintained in culture and may no longer faithfully reflect their original *in vivo* characteristics. Thus, we studied patient plasma in a correlative study. Our study using patient plasma supports this hypothesis; in sEVs from patients with more aggressive cancers, only the ST6GAL1 membrane-bound form is detected while both forms are expressed in sEVs from patients with less aggressive cancers. These findings align with a recent study showing that PrCa progression and therapy resistance correlate with changes in glycosylation patterns [48]. Both forms are also detected in healthy donor plasma sEVs. However, they float in lower-density fractions, where sEV markers co-fractionate, as well as in fractions where sEV markers are undetectable.

It should be noted that the difference in ST6GAL1 levels in DU145, PC3 and C4-2B cells may also be a result of the fact that DU145 and PC3 are androgen receptor (AR)-negative cell lines as compared to C4-2B which are AR-positive cells. Although AR promotes wide expression of ST6GAL1, as has been previously proposed [49], sEVs packaging is not dependent on AR [50].

Both membrane-bound and soluble forms of ST6GAL1 are active; thus, their activity may contribute to a malignant phenotype in an autocrine and paracrine manner. Our analysis provides evidence that the ST6GAL1 soluble form is transferred via sEVs to cancer cells and that the neighboring cancer cells may be the active recipient cells; however, it cannot be excluded that the sEVs themselves or cells (fibroblasts and immune cells) in the TME may utilize ST6GAL1. In fact, α2,6 sialylation is a key mechanism for polarizing T cells helper 2 responses [8], reprogramming tumor-associated endothelial cells [9] and rewiring the function of myeloid-derived suppressor cells [51] by modulating sensitivity to Galectin-1, which promotes immunosuppression, angiogenesis and metastasis through binding to different glycosylated receptors, including integrins [52]. Thus, the transfer of ST6GAL1 to immune or endothelial cells may also contribute to the cellular repertoire of the prostate TME by modulating different processes. In PrCa, Galectin-1 expression correlates with more advanced lesions in primary tumors and compared to other members of the family, it is the most highly expressed in LNCaP, a hormone-responsive cell line, as well as in the castrate-resistant 22Rv1 and PC3 cell lines [53]. Since Galectin-1-glycan lattice formation is sensitive to α2,6 sialylation [51], ST6GAL1 transfer to tumor-associated immune cells may outcompete the PD-1/PDL1 axis to foster immunosuppressive TME. Furthermore, this sialyltransferase may influence the signaling of the PD-1/PDL1 axis by remodeling the glycosylation profiles of these immune inhibitory checkpoints and modulating their signaling pattern, as recently proposed [54]. Finally, since Galectin-1 also contributes to PrCa progression [53], an interplay between ST6GAL1 transfer and Galectin-1 activity may modulate PrCa metastasis.

## Supporting information

S1 File Original images for blots. (PDF)

## References

[1] Stanczak MA, Rodrigues Mantuano N, Kirchhammer N, Sanin DE, Jacob F, Coelho R, et al. Targeting cancer glycosylation repolarizes tumor-associated macrophages allowing effective immune checkpoint blockade. Sci Transl Med. 2022;14(669):eabj1270. doi: 10.1126/scitranslmed.abj1270 36322632 PMC9812757

[2] Park J-J, Lee M. Increasing the α 2, 6 sialylation of glycoproteins may contribute to metastatic spread and therapeutic resistance in colorectal cancer. Gut Liver. 2013;7(6):629–41. doi: 10.5009/gnl.2013.7.6.629 24312702 PMC3848550

[3] Munkley J. Aberrant Sialylation in Cancer: Therapeutic Opportunities. Cancers (Basel). 2022;14(17):4248. doi: 10.3390/cancers14174248 36077781 PMC9454432

[4] Swindall AF, Londoño-Joshi AI, Schultz MJ, Fineberg N, Buchsbaum DJ, Bellis SL. ST6Gal-I protein expression is upregulated in human epithelial tumors and correlates with stem cell markers in normal tissues and colon cancer cell lines. Cancer Res. 2013;73(7):2368–78. doi: 10.1158/0008-5472.CAN-12-3424 23358684 PMC4038408

[5] Garnham R, Scott E, Livermore KE, Munkley J. ST6GAL1: A key player in cancer. Oncol Lett. 2019;18(2):983–9. doi: 10.3892/ol.2019.10458 31423157 PMC6607188

[6] Bresalier RS, Rockwell RW, Dahiya R, Duh QY, Kim YS. Cell surface sialoprotein alterations in metastatic murine colon cancer cell lines selected in an animal model for colon cancer metastasis. Cancer Res. 1990;50(4):1299–307. 2297775

[7] Bhalerao N, Chakraborty A, Marciel MP, Hwang J, Britain CM, Silva AD, et al. ST6GAL1 sialyltransferase promotes acinar to ductal metaplasia and pancreatic cancer progression. JCI Insight. 2023;8(19):e161563. doi: 10.1172/jci.insight.161563 37643018 PMC10619436

[8] Toscano MA, Bianco GA, Ilarregui JM, Croci DO, Correale J, Hernandez JD, et al. Differential glycosylation of TH1, TH2 and TH-17 effector cells selectively regulates susceptibility to cell death. Nat Immunol. 2007;8(8):825–34. doi: 10.1038/ni1482 17589510

[9] Croci DO, Cerliani JP, Dalotto-Moreno T, Méndez-Huergo SP, Mascanfroni ID, Dergan-Dylon S, et al. Glycosylation-dependent lectin-receptor interactions preserve angiogenesis in anti-VEGF refractory tumors. Cell. 2014;156(4):744–58. doi: 10.1016/j.cell.2014.01.043 24529377

[10] Zhuo Y, Chammas R, Bellis SL. Sialylation of beta1 integrins blocks cell adhesion to galectin-3 and protects cells against galectin-3-induced apoptosis. J Biol Chem. 2008;283(32):22177–85. doi: 10.1074/jbc.M8000015200 18676377 PMC2494929

[11] Scott E, Archer Goode E, Garnham R, Hodgson K, Orozco-Moreno M, Turner H, et al. ST6GAL1-mediated aberrant sialylation promotes prostate cancer progression. J Pathol. 2023;261(1):71–84. doi: 10.1002/path.6152 37550801

[12] Wei A, Fan B, Zhao Y, Zhang H, Wang L, Yu X, et al. ST6Gal-I overexpression facilitates prostate cancer progression via the PI3K/Akt/GSK-3β/β-catenin signaling pathway. Oncotarget. 2016;7(40):65374–88. doi: 10.18632/oncotarget.11699 27588482 PMC5323162

[13] Hodgson K, Orozco-Moreno M, Goode EA, Fisher M, Garnham R, Beatson R, et al. Sialic acid blockade inhibits the metastatic spread of prostate cancer to bone. EBioMedicine. 2024;104:105163. doi: 10.1016/j.ebiom.2024.105163 38772281 PMC11134892

[14] Voss M. Proteolytic cleavage of Golgi glycosyltransferases by SPPL3 and other proteases and its implications for cellular glycosylation. Biochim Biophys Acta Gen Subj. 2024;1868(10):130668. doi: 10.1016/j.bbagen.2024.130668 38992482

[15] Hait NC, Maiti A, Wu R, Andersen VL, Hsu C-C, Wu Y, et al. Extracellular sialyltransferase st6gal1 in breast tumor cell growth and invasiveness. Cancer Gene Ther. 2022;29(11):1662–75. doi: 10.1038/s41417-022-00485-y 35676533 PMC9663294

[16] Kitazume S, Tachida Y, Oka R, Shirotani K, Saido TC, Hashimoto Y. Alzheimer’s beta-secretase, beta-site amyloid precursor protein-cleaving enzyme, is responsible for cleavage secretion of a Golgi-resident sialyltransferase. Proc Natl Acad Sci U S A. 2001;98(24):13554–9. doi: 10.1073/pnas.241509198 11698669 PMC61079

[17] Irons EE, Gc S, Lau JTY. Sialic acid in the regulation of blood cell production, differentiation and turnover. Immunology. 2024;172(4):517–32. doi: 10.1111/imm.13780 38503445 PMC11223974

[18] Kaplan HA, Woloski BM, Hellman M, Jamieson JC. Studies on the effect of inflammation on rat liver and serum sialyltransferase. Evidence that inflammation causes release of Gal beta 1 leads to 4GlcNAc alpha 2 leads to 6 sialyltransferase from liver. J Biol Chem. 1983;258(19):11505–9. 6413502

[19] Kitazume S, Oka R, Ogawa K, Futakawa S, Hagiwara Y, Takikawa H, et al. Molecular insights into beta-galactoside alpha2,6-sialyltransferase secretion in vivo. Glycobiology. 2009;19(5):479–87. doi: 10.1093/glycob/cwp003 19150807

[20] Lee-Sundlov MM, Ashline DJ, Hanneman AJ, Grozovsky R, Reinhold VN, Hoffmeister KM, et al. Circulating blood and platelets supply glycosyltransferases that enable extrinsic extracellular glycosylation. Glycobiology. 2017;27(2):188–98. doi: 10.1093/glycob/cww108 27798070 PMC5224594

[21] Lee MM, Nasirikenari M, Manhardt CT, Ashline DJ, Hanneman AJ, Reinhold VN, et al. Platelets support extracellular sialylation by supplying the sugar donor substrate. J Biol Chem. 2014;289(13):8742–8. doi: 10.1074/jbc.C113.546713 24550397 PMC3979374

[22] Manhardt CT, Punch PR, Dougher CWL, Lau JTY. Extrinsic sialylation is dynamically regulated by systemic triggers in vivo. J Biol Chem. 2017;292(33):13514–20. doi: 10.1074/jbc.C117.795138 28717006 PMC5566511

[23] Rusiniak ME, Punch PR, Hait NC, Maiti A, Burns RT, Chapla D, et al. Extracellular ST6GAL1 regulates monocyte-macrophage development and survival. Glycobiology. 2022;32(8):701–11. doi: 10.1093/glycob/cwac032 35661210 PMC9280526

[24] Irons EE, Lee-Sundlov MM, Zhu Y, Neelamegham S, Hoffmeister KM, Lau JT. B cells suppress medullary granulopoiesis by an extracellular glycosylation-dependent mechanism. Elife. 2019;8:e47328. doi: 10.7554/eLife.47328 31408003 PMC6713473

[25] Dougher CWL, Buffone A Jr, Nemeth MJ, Nasirikenari M, Irons EE, Bogner PN, et al. The blood-borne sialyltransferase ST6Gal-1 is a negative systemic regulator of granulopoiesis. J Leukoc Biol. 2017;102(2):507–16. doi: 10.1189/jlb.3A1216-538RR 28550122 PMC5505748

[26] Zhang Q, Higginbotham JN, Jeppesen DK, Yang Y-P, Li W, McKinley ET, et al. Transfer of Functional Cargo in Exomeres. Cell Rep. 2019;27(3):940-954.e6. doi: 10.1016/j.celrep.2019.01.009 30956133 PMC6559347

[27] Goel HL, Sayeed A, Breen M, Zarif MJ, Garlick DS, Leav I, et al. β1 integrins mediate resistance to ionizing radiation in vivo by inhibiting c-Jun amino terminal kinase 1. J Cell Physiol. 2013;228(7):1601–9. doi: 10.1002/jcp.24323 23359252 PMC3749928

[28] Quaglia F, Krishn SR, Sossey-Alaoui K, Rana PS, Pluskota E, Park PH, et al. The NOGO receptor NgR2, a novel αVβ3 integrin effector, induces neuroendocrine differentiation in prostate cancer. Sci Rep. 2022;12(1):18879. doi: 10.1038/s41598-022-21711-5 36344556 PMC9640716

[29] Quaglia F, Krishn SR, Daaboul GG, Sarker S, Pippa R, Domingo-Domenech J, et al. Small extracellular vesicles modulated by αVβ3 integrin induce neuroendocrine differentiation in recipient cancer cells. J Extracell Vesicles. 2020;9(1):1761072. doi: 10.1080/20013078.2020.1761072 32922691 PMC7448905

[30] Salem I, Naranjo NM, Singh A, DeRita R, Krishn SR, Sirman LS, et al. Methods for extracellular vesicle isolation from cancer cells. Cancer Drug Resist. 2020;3(3):371–84. doi: 10.20517/cdr.2019.118 33062957 PMC7556721

[31] Naranjo NM, Salem I, Harris MA, Languino LR. IFIT3 (interferon induced protein with tetratricopeptide repeats 3) modulates STAT1 expression in small extracellular vesicles. Biochem J. 2021;478(21):3905–21. doi: 10.1042/BCJ20210580 34622927 PMC9121857

[32] Verrillo CE, Quaglia F, Shields CD, Lin S, Kossenkov AV, Tang H-Y, et al. Expression of the αVβ3 integrin affects prostate cancer sEV cargo and density and promotes sEV pro-tumorigenic activity in vivo through a GPI-anchored receptor, NgR2. J Extracell Vesicles. 2024;13(8):e12482. doi: 10.1002/jev2.12482 39105261 PMC11301027

[33] Théry C, Ostrowski M, Segura E. Membrane vesicles as conveyors of immune responses. Nat Rev Immunol. 2009;9(8):581–93. doi: 10.1038/nri2567 19498381

[34] Onódi Z, Pelyhe C, Terézia Nagy C, Brenner GB, Almási L, Kittel Á, et al. Isolation of High-Purity Extracellular Vesicles by the Combination of Iodixanol Density Gradient Ultracentrifugation and Bind-Elute Chromatography From Blood Plasma. Front Physiol. 2018;9:1479. doi: 10.3389/fphys.2018.01479 30405435 PMC6206048

[35] Iwai K, Minamisawa T, Suga K, Yajima Y, Shiba K. Isolation of human salivary extracellular vesicles by iodixanol density gradient ultracentrifugation and their characterizations. J Extracell Vesicles. 2016;5:30829. doi: 10.3402/jev.v5.30829 27193612 PMC4871899

[36] Kowal J, Arras G, Colombo M, Jouve M, Morath JP, Primdal-Bengtson B, et al. Proteomic comparison defines novel markers to characterize heterogeneous populations of extracellular vesicle subtypes. Proc Natl Acad Sci U S A. 2016;113(8):E968-77. doi: 10.1073/pnas.1521230113 26858453 PMC4776515

[37] Thalmann GN, Anezinis PE, Chang SM, Zhau HE, Kim EE, Hopwood VL, et al. Androgen-independent cancer progression and bone metastasis in the LNCaP model of human prostate cancer. Cancer Res. 1994;54(10):2577–81. 8168083

[38] Holdbrooks AT, Ankenbauer KE, Hwang J, Bellis SL. Regulation of inflammatory signaling by the ST6Gal-I sialyltransferase. PLoS One. 2020;15(11):e0241850. doi: 10.1371/journal.pone.0241850 33166339 PMC7652342

[39] Voeks DJ, Martiniello-Wilks R, Russell PJ. Derivation of MPR and TRAMP models of prostate cancer and prostate cancer metastasis for evaluation of therapeutic strategies. Urol Oncol. 2002;7(3):111–8. doi: 10.1016/s1078-1439(01)00180-6 12474544

[40] Britain CM, Holdbrooks AT, Anderson JC, Willey CD, Bellis SL. Sialylation of EGFR by the ST6Gal-I sialyltransferase promotes EGFR activation and resistance to gefitinib-mediated cell death. J Ovarian Res. 2018;11(1):12. doi: 10.1186/s13048-018-0385-0 29402301 PMC5800010

[41] Holdbrooks AT, Britain CM, Bellis SL. ST6Gal-I sialyltransferase promotes tumor necrosis factor (TNF)-mediated cancer cell survival via sialylation of the TNF receptor 1 (TNFR1) death receptor. J Biol Chem. 2018;293(5):1610–22. doi: 10.1074/jbc.M117.801480 29233887 PMC5798293

[42] Gc S, Tuy K, Rickenbacker L, Jones R, Chakraborty A, Miller CR, et al. α2,6 Sialylation mediated by ST6GAL1 promotes glioblastoma growth. JCI Insight. 2022;7(21):e158799. doi: 10.1172/jci.insight.158799 36345944 PMC9675560

[43] Pocheć E, Bubka M, Rydlewska M, Janik M, Pokrywka M, Lityńska A. Aberrant glycosylation of αvβ3 integrin is associated with melanoma progression. Anticancer Res. 2015;35(4):2093–103. 25862865

[44] Kremser ME, Przybyło M, Hoja-Łukowicz D, Pocheć E, Amoresano A, Carpentieri A, et al. Characterisation of alpha3beta1 and alpha(v)beta3 integrin N-oligosaccharides in metastatic melanoma WM9 and WM239 cell lines. Biochim Biophys Acta. 2008;1780(12):1421–31. doi: 10.1016/j.bbagen.2008.07.011 18755246

[45] Venkatesh K, Chivatakarn O, Lee H, Joshi PS, Kantor DB, Newman BA, et al. The Nogo-66 receptor homolog NgR2 is a sialic acid-dependent receptor selective for myelin-associated glycoprotein. J Neurosci. 2005;25(4):808–22. doi: 10.1523/JNEUROSCI.4464-04.2005 15673660 PMC6725623

[46] Pendiuk Goncalves J, Cruz Villarreal J, Walker SA, Tan XNS, Borges C, Wolfram J. High-throughput analysis of glycan sorting into extracellular vesicles. Biochim Biophys Acta Mol Cell Res. 2024;1871(2):119641. doi: 10.1016/j.bbamcr.2023.119641 37996057

[47] Shupp AB, Neupane M, Agostini LC, Ning G, Brody JR, Bussard KM. Stromal-Derived Extracellular Vesicles Suppress Proliferation of Bone Metastatic Cancer Cells Mediated by ERK2. Mol Cancer Res. 2021;19(10):1763–77. doi: 10.1158/1541-7786.MCR-20-0981 34021072 PMC8492519

[48] Butler W, McDowell C, Yang Q, He Y, Zhao Y, Hauck JS, et al. Rewiring of the N-Glycome with prostate cancer progression and therapy resistance. NPJ Precis Oncol. 2023;7(1):22. doi: 10.1038/s41698-023-00363-2 36828904 PMC9958128

[49] Munkley J, Vodak D, Livermore KE, James K, Wilson BT, Knight B, et al. Glycosylation is an Androgen-Regulated Process Essential for Prostate Cancer Cell Viability. EBioMedicine. 2016;8:103–16. doi: 10.1016/j.ebiom.2016.04.018 27428423 PMC4919605

[50] Bizzaro CL, Bach CA, Santos RA, Verrillo CE, Naranjo NM, Chaudhari I, et al. Exploring STEAP1 Expression in Prostate Cancer Cells in Response to Androgen Deprivation and in Small Extracellular Vesicles. Mol Cancer Res. 2025;23(6):542–52. doi: 10.1158/1541-7786.MCR-24-0903 40287951 PMC12133427

[51] Blidner AG, Bach CA, García PA, Merlo JP, Cagnoni AJ, Bannoud N, et al. Glycosylation-driven programs coordinate immunoregulatory and pro-angiogenic functions of myeloid-derived suppressor cells. Immunity. 2025;58(6):1553-1571.e8. doi: 10.1016/j.immuni.2025.04.027 40381622

[52] Troncoso MF, Elola MT, Blidner AG, Sarrias L, Espelt MV, Rabinovich GA. The universe of galectin-binding partners and their functions in health and disease. J Biol Chem. 2023;299(12):105400. doi: 10.1016/j.jbc.2023.105400 37898403 PMC10696404

[53] Laderach DJ, Gentilini LD, Giribaldi L, Delgado VC, Nugnes L, Croci DO, et al. A unique galectin signature in human prostate cancer progression suggests galectin-1 as a key target for treatment of advanced disease. Cancer Res. 2013;73(1):86–96. doi: 10.1158/0008-5472.CAN-12-1260 23108139

[54] Lee T-A, Tsai E-Y, Liu S-H, Chou W-C, Hsu Hung S-D, Chang C-Y, et al. Regulation of PD-L1 glycosylation and advances in cancer immunotherapy. Cancer Lett. 2025;612:217498. doi: 10.1016/j.canlet.2025.217498 39855377

