# Supplementary material for: A novel sialylation pathway mediated by extracellular vesicles in aggressive prostate cancer
Source: PLoS One. 2025 Sep 12;20(9):e0329014. doi: 10.1371/journal.pone.0329014 (PMC12431281; doi:10.1371/journal.pone.0329014)

Figure 2A

Top panel

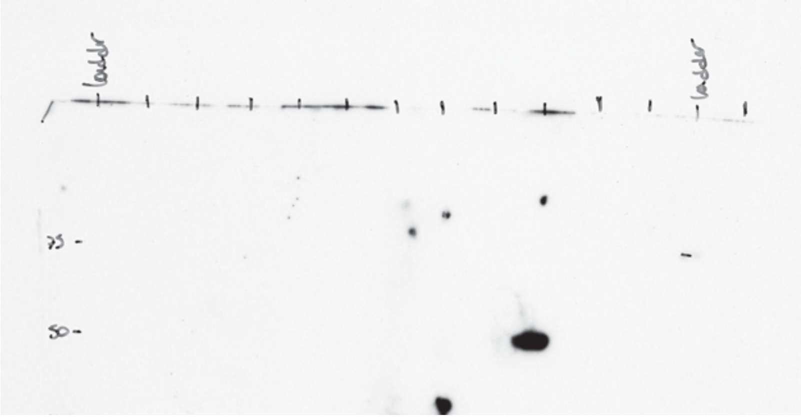

ST6GAL1

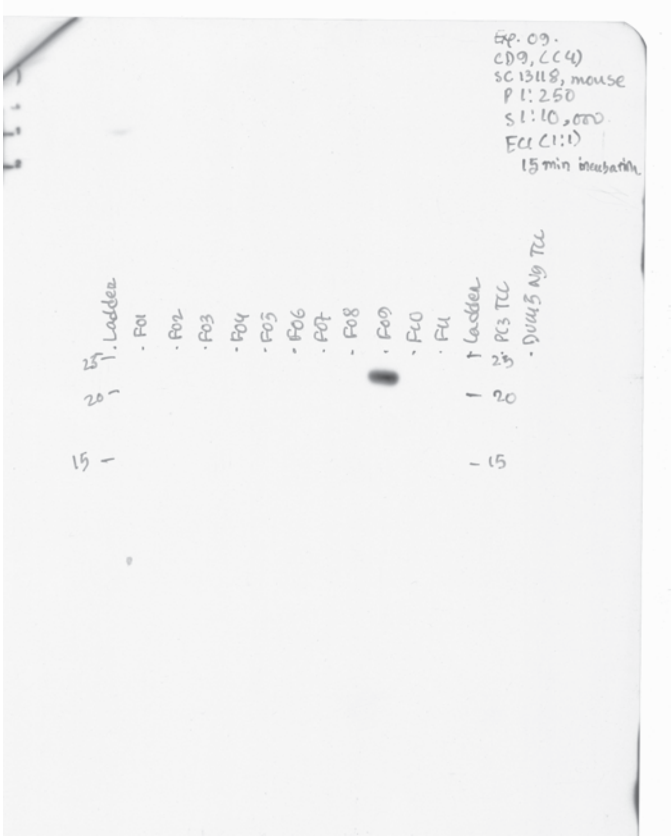

CD9

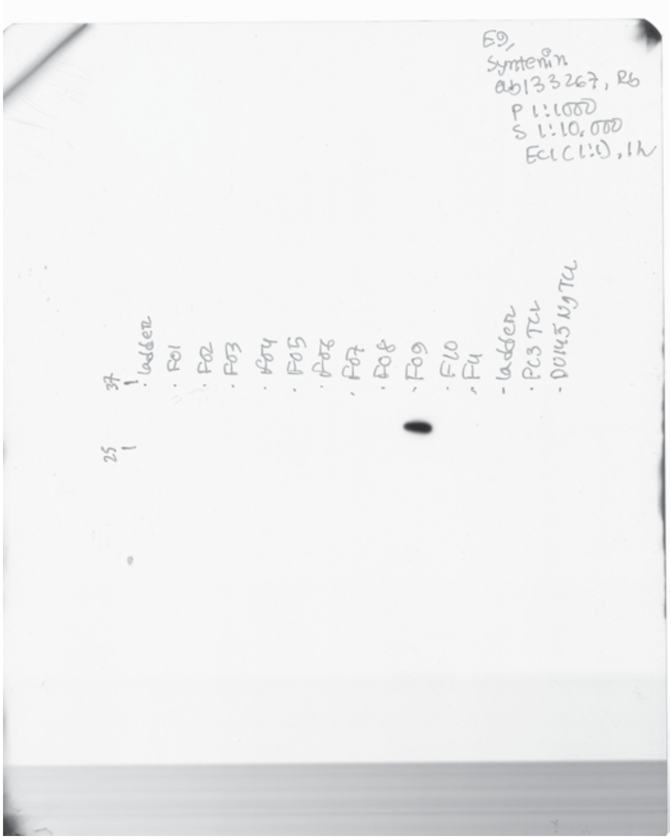

Syntenin

Figure 2A

Bottom panel

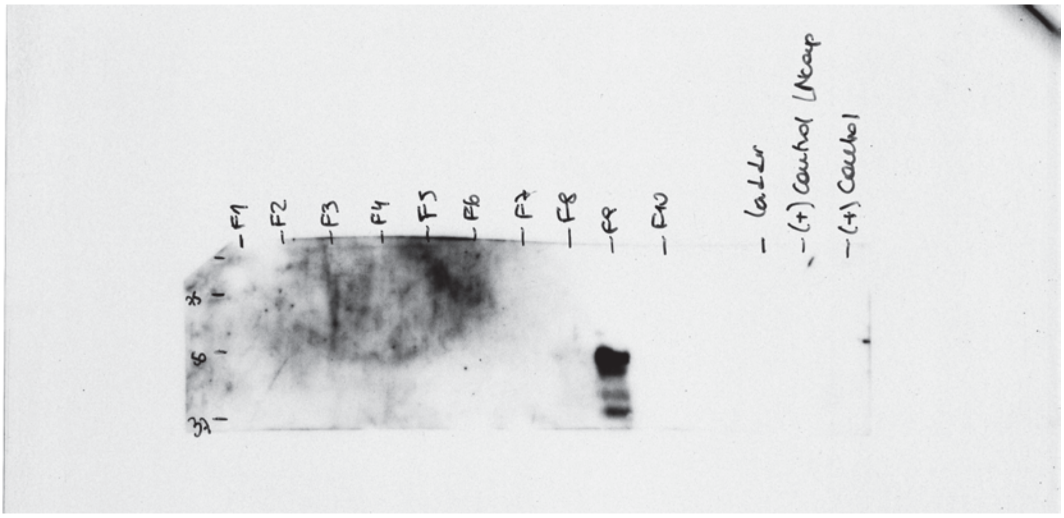

ST6GAL1

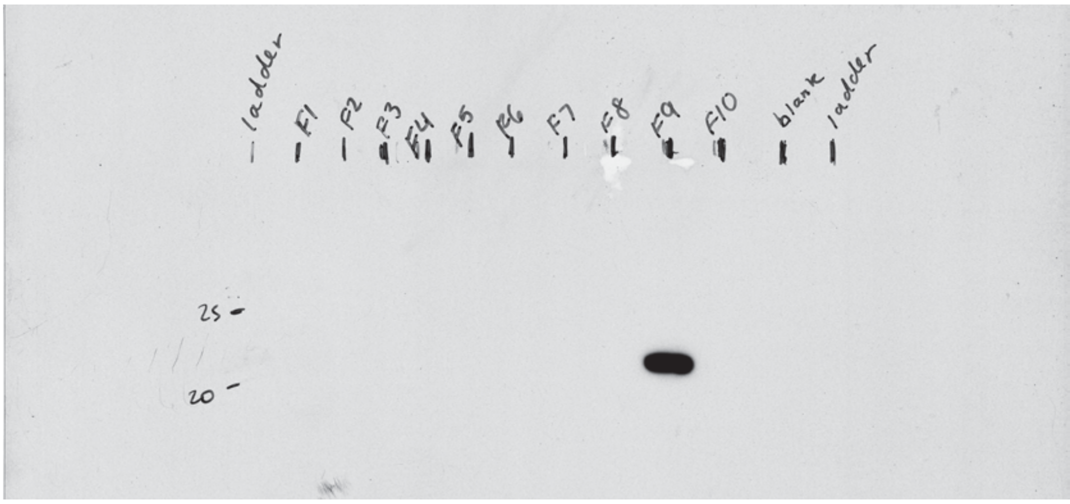

CD9

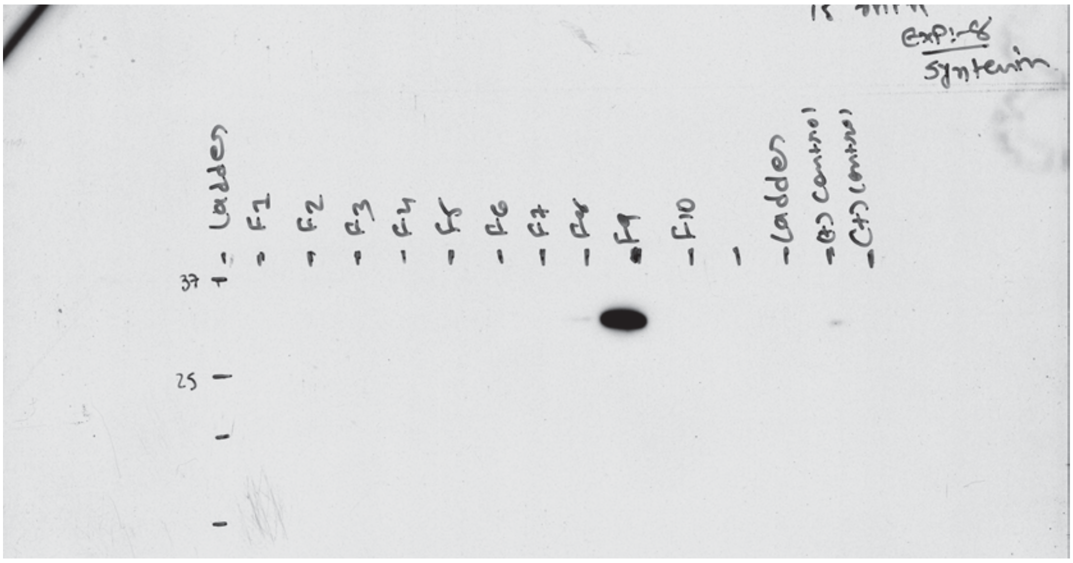

Syntenin

Figure 2B

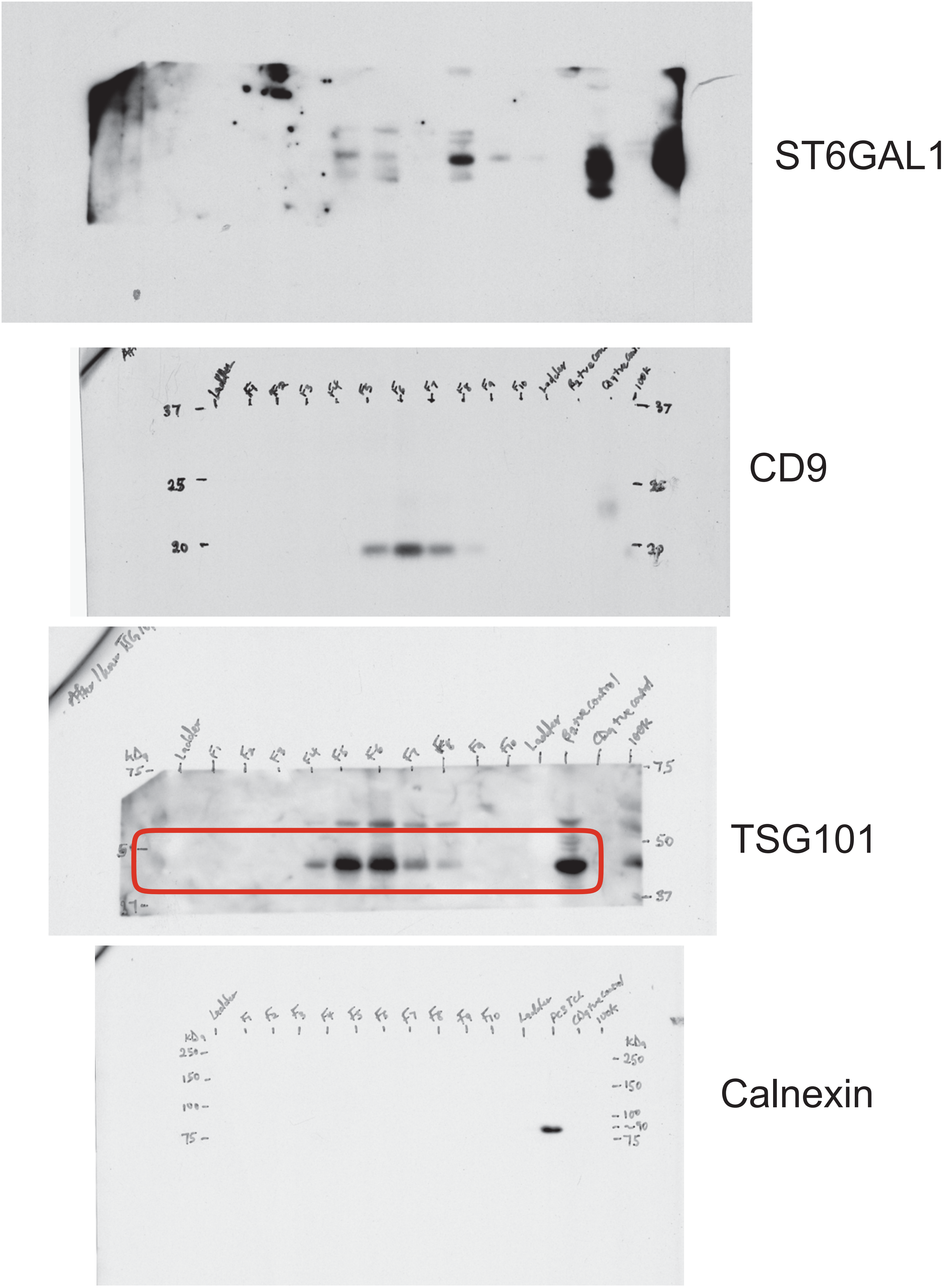

Figure 2C

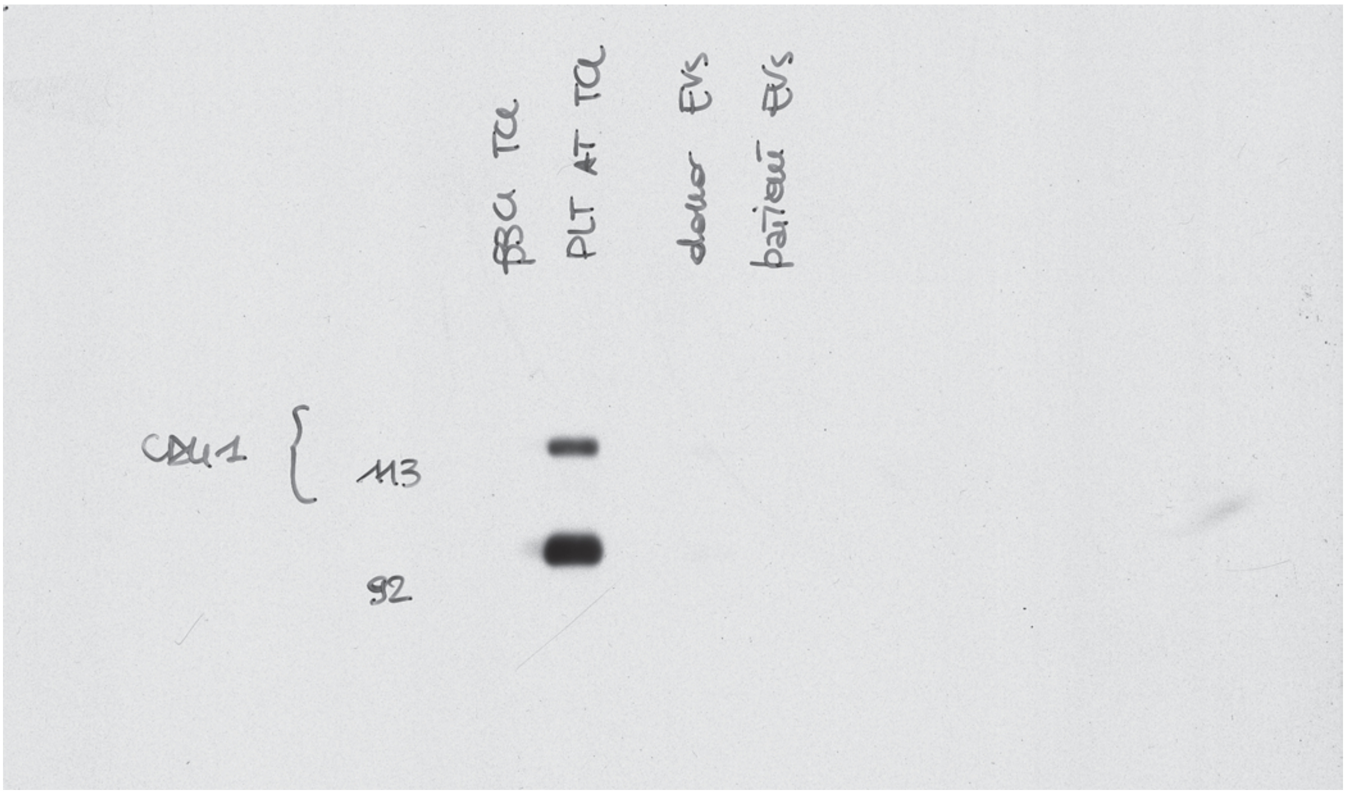

CD41

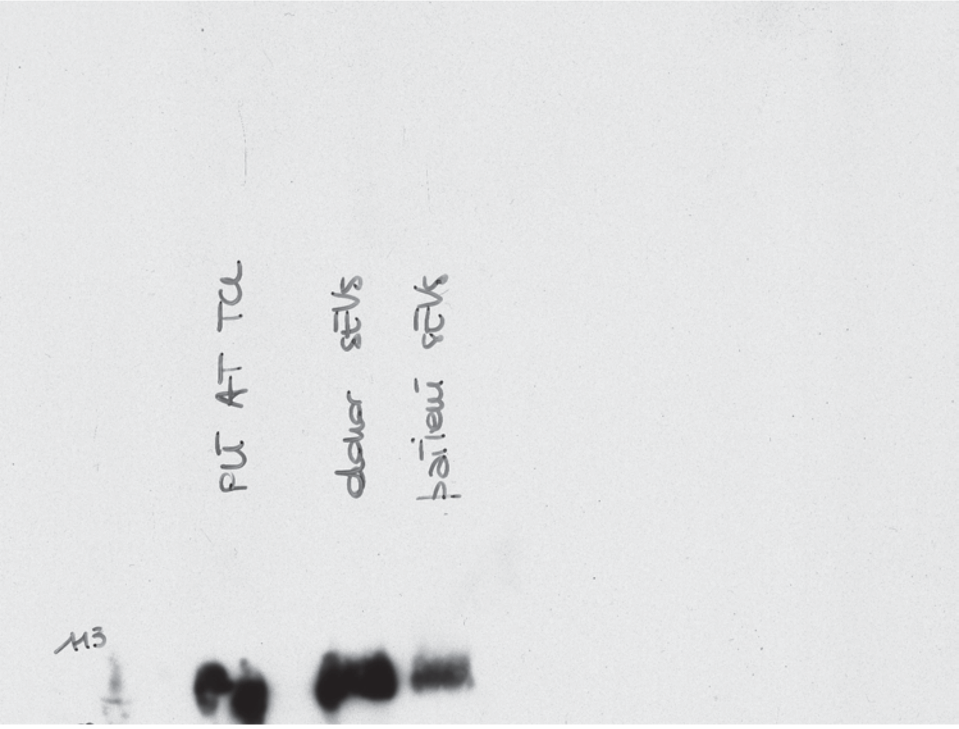

Beta3

Figure 3

ST6GAL1 in total cell lysate

Lighter

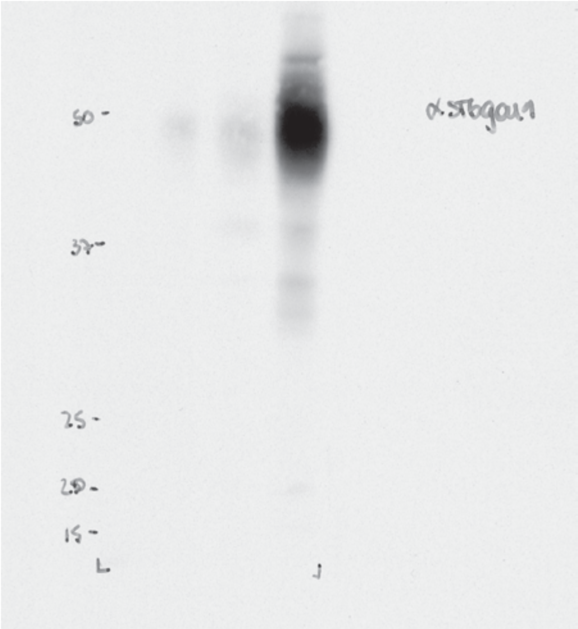

Darker

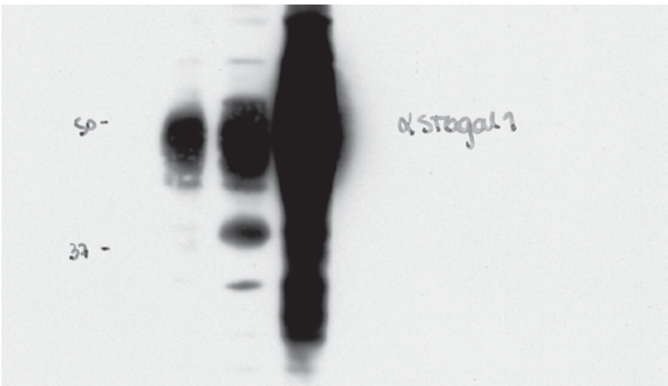

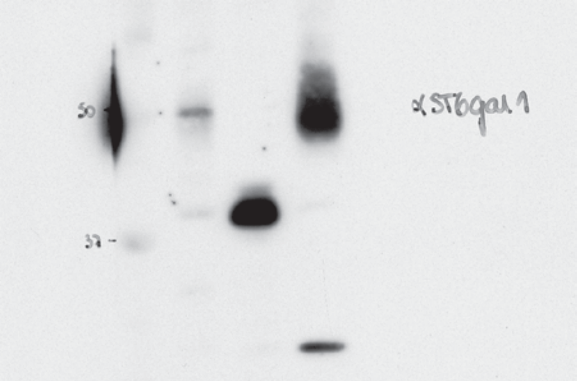

ST6GAL1 EVs

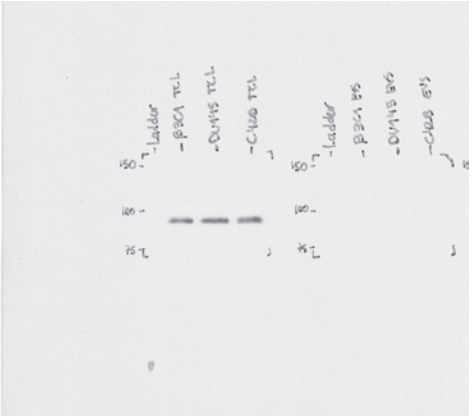

CNX

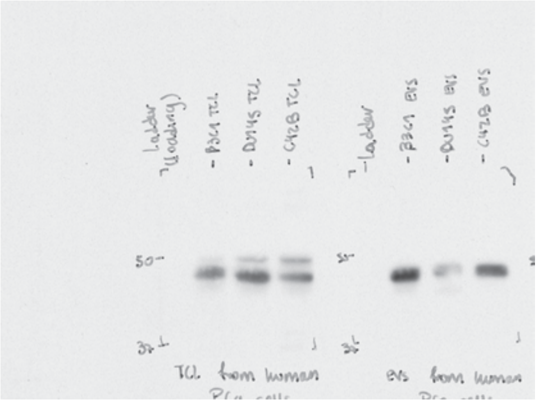

TSG101

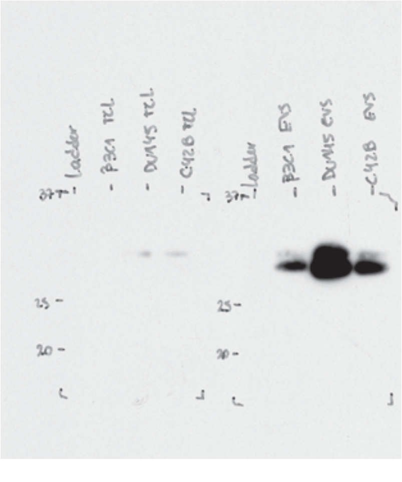

Syntenin

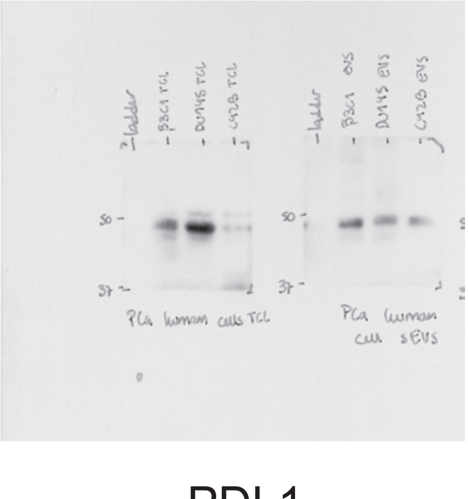

PDL1

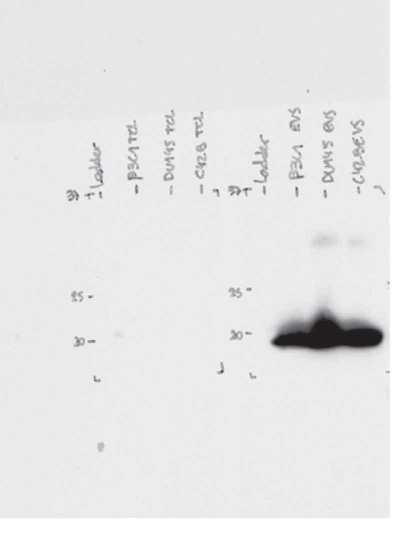

CD81

Figure 4A

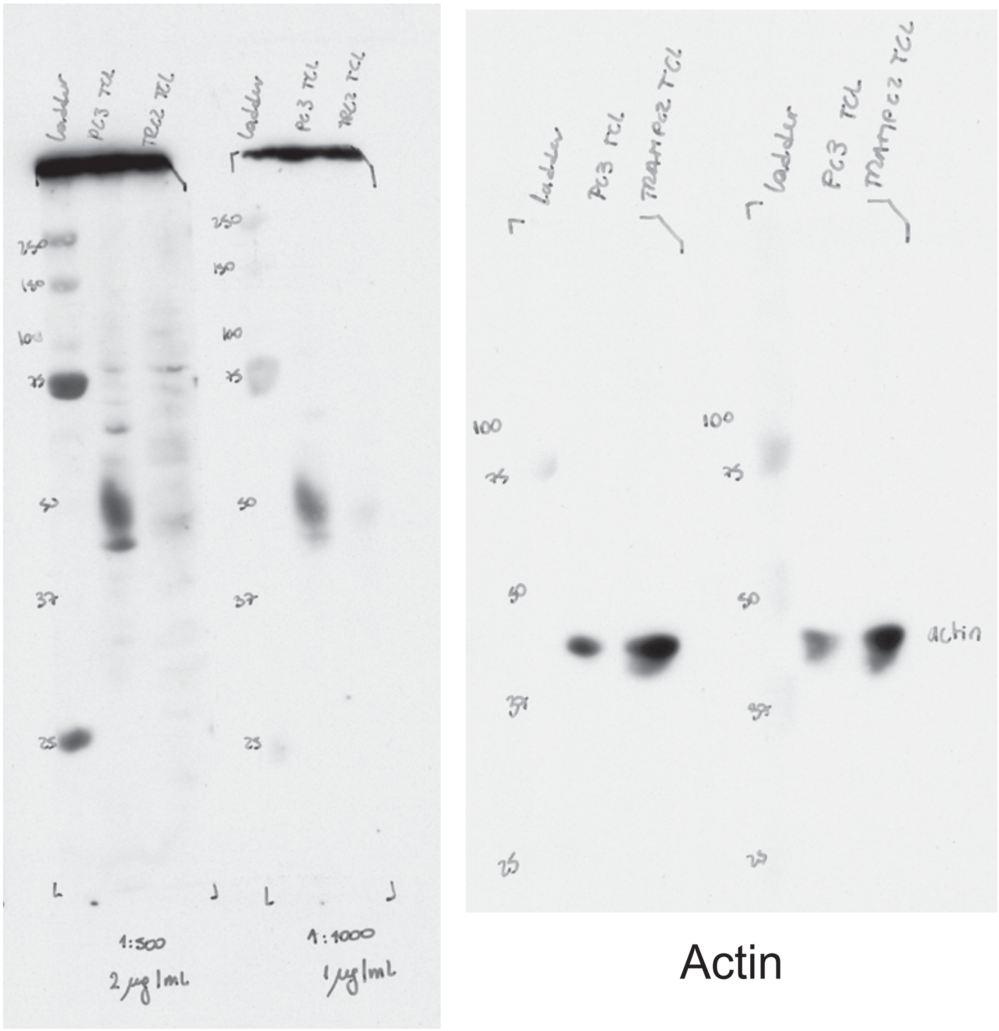

ST6GAL1

Actin

Figure 4B

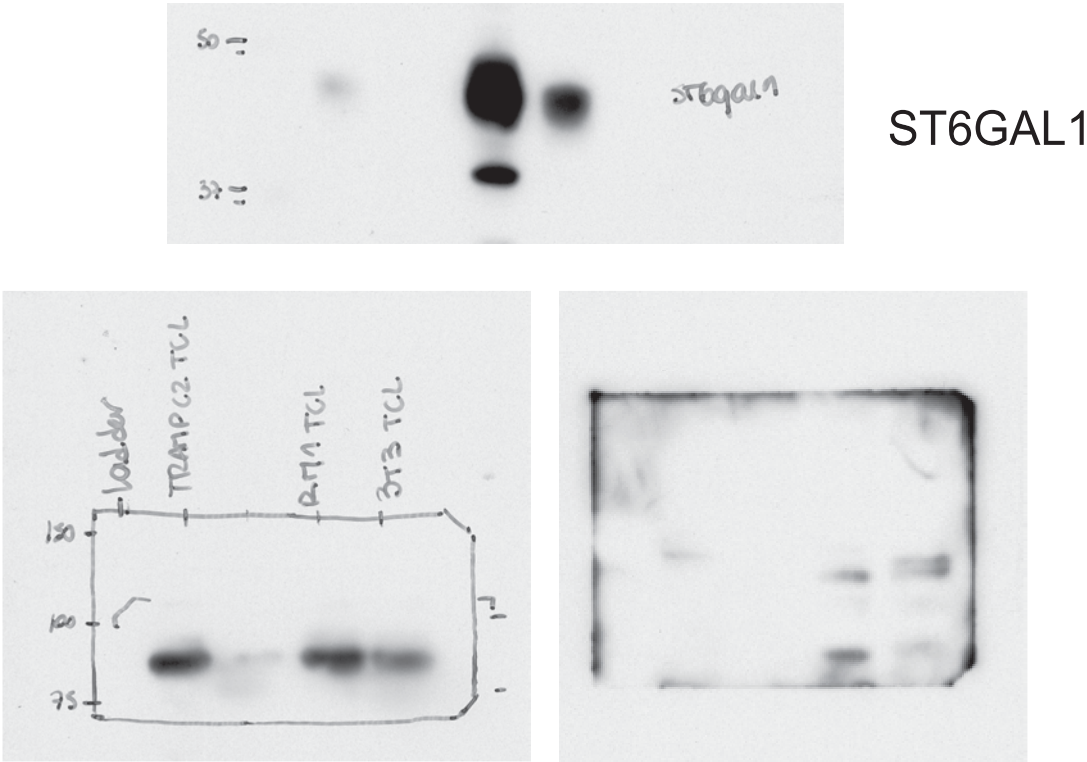

ST6GAL1

CNX

PDL1

Figure 5A

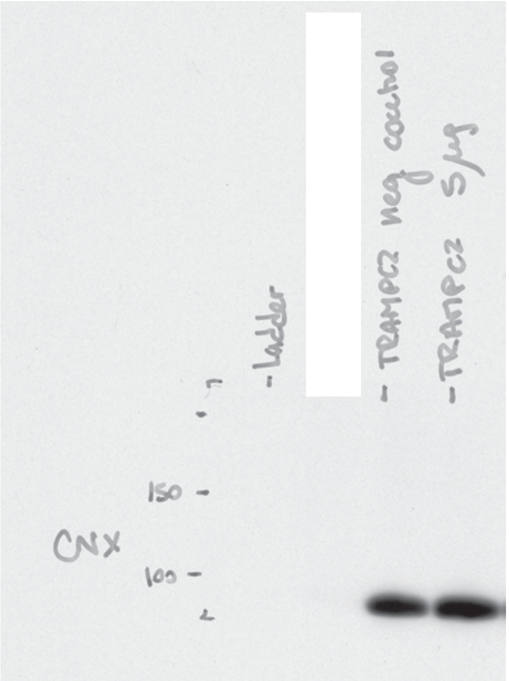

CNX

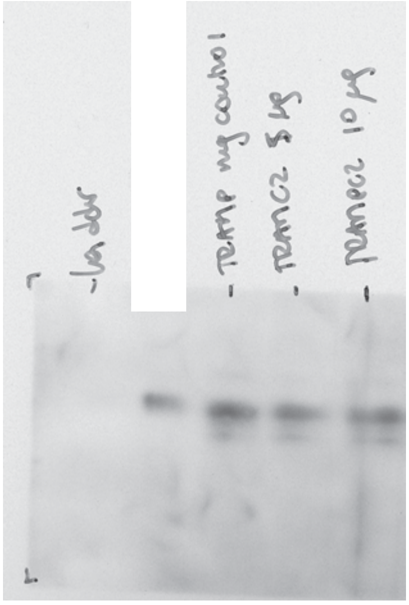

TSG101

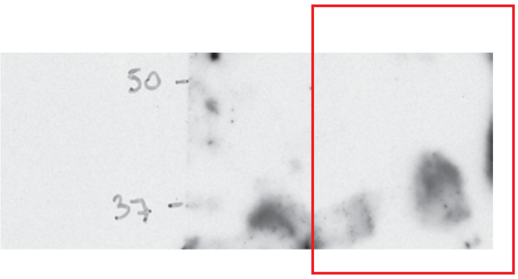

ST6GAL1

Figure 5B

ST6GAL1

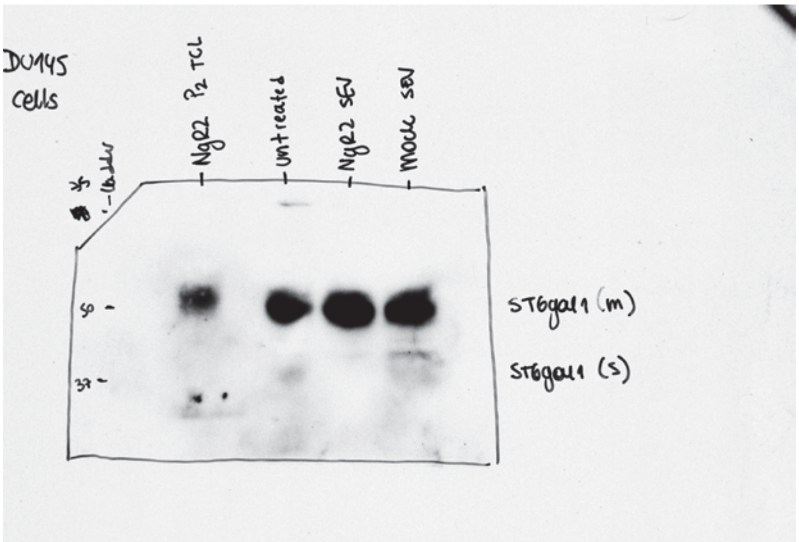

FAK

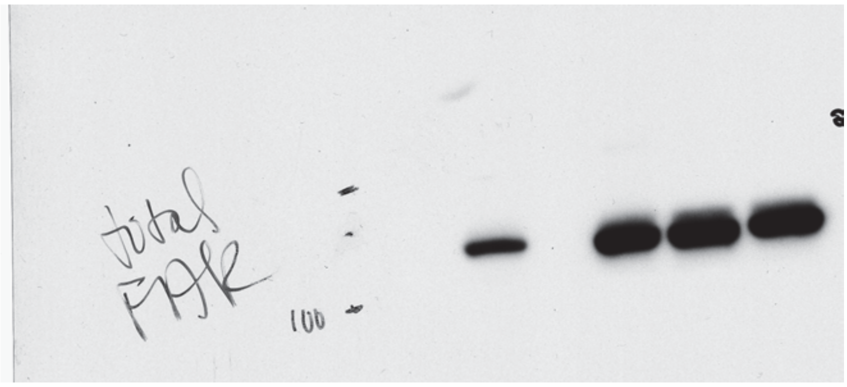

Supplement: S1 File — (PDF) [file pone.0329014.s001.pdf]
